# Prostanoid Receptors Involved in Regulation of the Beating Rate of Neonatal Rat Cardiomyocytes

**DOI:** 10.1371/journal.pone.0045273

**Published:** 2012-09-12

**Authors:** Hakima Mechiche, Stanislas Grassin-Delyle, Arnaud Robinet, Pierre Nazeyrollas, Philippe Devillier

**Affiliations:** 1 Laboratory of Cardiovascular Pharmacology, Université Champagne Ardennes, Reims, France; 2 UPRES EA 220, Université Versailles Saint-Quentin en Yvelines, Hôpital Foch, Suresnes, France; Medical School of Hannover, United States of America

## Abstract

Although prostanoids are known to be involved in regulation of the spontaneous beating rate of cultured neonatal rat cardiomyocytes, the various subtypes of prostanoid receptors have not been investigated in detail. In our experiments, prostaglandin (PG)F_2α_ and prostanoid FP receptor agonists (fluprostenol, latanoprost and cloprostenol) produced a decrease in the beating rate. Two prostanoid IP receptor agonists (iloprost and beraprost) induced first a marked drop in the beating rate and then definitive abrogation of beating. In contrast, the prostanoid DP receptor agonists (PGD_2_ and BW245C) and TP receptor agonists (U-46619) produced increases in the beating rate. Sulprostone (a prostanoid EP_1_ and EP_3_ receptor agonist) induced marked increases in the beating rate, which were suppressed by SC-19220 (a selective prostanoid EP_1_ antagonist). Butaprost (a selective prostanoid EP_2_ receptor agonist), misoprostol (a prostanoid EP_2_ and EP_3_ receptor agonist), 11-deoxy-PGE_1_ (a prostanoid EP_2_, EP_3_ and EP_4_ receptor agonist) did not alter the beating rate. Our results strongly suggest that prostanoid EP_1_ receptors are involved in positive regulation of the beating rate. Prostanoid EP_1_ receptor expression was confirmed by western blotting with a selective antibody. Hence, neonatal rat cardiomyocytes express both prostanoid IP and FP receptors (which negatively regulate the spontaneous beating rate) and prostanoid TP, DP_1_ and EP_1_ receptors (which positively regulate the spontaneous beating rate).

## Introduction

Prostanoids exert a wide variety of effects on cardiac tissue [1]. The prostanoid receptors include the DP, EP, FP, IP_1_ and TP receptors [2], [3], which preferentially bind prostaglandin D (PGD), PGE, PGF, PGI and thromboxane A (TXA), respectively, and belong to the G-protein-coupled receptor superfamily [4]. The prostanoid EP receptor type has EP_1_, EP_2_, EP_3_ and EP_4_ subtypes and the DP receptor type has DP_1_ and DP_2_ subtypes [4]. Although endogenous prostanoids tend to bind most strongly to a given prostanoid receptor class, there is a marked degree of cross-reactivity between these ligands and the entire receptor family [2], [5]. This taxonomy has only been described fairly recently, following the availability of selective agonists and, to lesser degree, a few selective antagonists [6]. Molecular biology techniques have confirmed this pharmacological classification via the cloning of cDNAs for representatives of each type of prostanoid receptor in a number of species (including the human, the rat and the mouse [2], [4], [7], [8].

In the heart, prostanoid synthesis is enhanced after (i) ischemia produced *in situ* by coronary artery ligation or (ii) exposure to inflammatory cytokines [1], [9], [10]. The cardiac production of prostanoids appears to be involved in ischemic preconditioning's protective effect against reperfusion-induced tachyarrhythmia in the isolated rat heart [11], [12] and mediates inflammation-associated tachycardia in mice [10]. Furthermore, the fact that endogenous prostanoids can alter the beating rate of neonatal rat cardiomyocytes [13] provides additional insight into the relationship between these compounds and cardiac arrhythmia. Neonatal rat cardiomyocytes - which beat spontaneously, synchronously and rhythmically - provide a useful preparation for assessing the direct chronotropic and arrythmogenic effects of pharmacological agents and avoid the possible confounding effects of neurogenic or circulating humoral factors [13], [14]. However, the receptors involved in the prostanoids' chronotropic effects in this preparation have not been systematically investigated by applying selective, synthetic prostanoid receptor agonists or antagonists.

The main objective of the present study was thus to characterize the prostanoid receptor types and subtypes involved in regulation of the spontaneous beating rate of neonatal rat cardiomyocytes. To that end, we mainly applied selected potent, prostanoid receptor agonists to the preparation.

## Materials and Methods

### Cell culture

This study was approved by the Faculty of Pharmacy and Medicine in Reims. Spontaneously beating neonatal rat cardiomyocyte cultures were obtained from 2- to 4-day-old Sprague-Dawley rats. The animal facilities and the staff involved in the animal experiments were accredited by the Veterinary Service of the French Ministry of Agriculture. The rats were deeply anesthetized with 50 mg/kg of sodium pentobarbital injected intraperitoneally. Briefly, heart tissue was washed with Eagle's minimum essential medium (MEM) (Sigma, St-Louis, MO, USA) containing 10% foetal calf serum (FCS) (DAP, Vogelgrun, France), 2 mM glutamine (Institut J. Boy, Reims, France), penicillin 1,000 U/ml (Specia, Paris, France) and streptomycin 0.1 mg/ml (Diamant, Puteaux, France) and buffered at pH 7.4 with sodium bicarbonate. Ventricular heart tissue was cut into small pieces, further washed with MEM and enzymatically digested for 15 minutes with 25 ml of phosphate-buffered saline with 40 U/ml collagenase (Sigma, St-Louis, MO, USA) at 37°C with constant stirring. The supernatant from the initial incubation was discarded, 25 ml of fresh enzyme solution were added and the incubation procedure was repeated six times. Subsequent supernatants were collected and centrifuged at 250 g for 5 min. The resulting cell pellets were resuspended in MEM at 37°C, pooled (3×10^5^ cells/ml) and seeded in 60 mm culture dishes (Corning, New York, USA) at a density of 1.8×10^6^ cells/dish. The cell cultures were incubated at 37°C in 95% air/5% CO_2_ for three days, with the medium changed daily. Most of the cultured cells (>90%) began to contract spontaneously within 24–48 h of plating. Confluent, spontaneously beating cells from day 3 of culture were used for all experiments, since homogenization of the beating rates was observed at day 3. Prior to experimental treatments, cell cultures were washed twice and incubated for 1 h with MEM containing 2% foetal calf serum and 1 µM indomethacin. This pretreatment was performed in order to avoid interference by endogenous prostanoids produced by the cardiomyocytes [9]. Indomethacin did not alter the beating rate, as previously shown by Li et al. [13]. All drug solutions were prepared daily.

### Measurement of the beating rate

The culture dish was placed on the heating stage of an inverted phase contrast microscope (model CK2 TR from Olympus Optical, Tokyo, Japan). The cells were magnified using a 20× objective and the image was monitored with a solid-state charge-coupled device camera (model 4712–7000 from Cohu, San Diego, CA, USA) attached to the microscope observation tube using an 6.7 coupler. The video output was connected to a television monitor (model VM-1221 from Hitachi Denshi, Tokyo, Japan).

The beating rate was determined simultaneously by two researchers, who measured the time required for 20 beats. Each researcher performed two independent measurements with a 30-second interval. At the end of each series of experiments, the time required for 20 beats was converted into a frequency per minute and the mean of the four determinations was calculated. The beating rate was measured every two minutes during the 10 minutes immediately preceding addition of the test compound.

The vials containing the test compound solutions were coded for random, blind application to the cardiomyocyte cultures. Two different methods were used. The first consisted of simply adding 100 µL of solution to the culture dish, in order to expose the cells to a concentration of 1 µM. The beating rate was then measured during the first minute and then every other minute over a 21-minute period. This screening method was used to identify compounds that caused significant, reproducible changes in the beating rate and to study the time course of these changes. This screening method was also used to study the responses to different concentrations of PGE_2_ and sulprostone. The second method consisted in exposing cell cultures to cumulative increases (from 1 nM to 1 µM to 1 log-increments) in the concentration of a selected compound. The concentration was increased every 7 minutes, since an optimal response was always observed within 5 minutes of addition. The beating rate was measured for 1 minute after each concentration change and then every other minute during each 7-minute interval. For experiments with the prostanoid EP_1_ receptor antagonist SC-19220, the compound was added to the culture dishes 15 minutes before addition of sulprostone. In all experiments, concentrations are quoted as the final molarity in the culture dishes. No more than two experiments were conducted with one agonist on dishes from the same culture preparation. For each series of experiments, control experiments were performed by applying the corresponding volumes of the compound's vehicle. A cell viability assay (trypan blue dye exclusion) was performed on at least two rat cardiomyocyte preparations at the end of experiments with vehicles or the compounds. Cell viability exceeded 90% in all cases. The stability of the beating frequency was checked on control dishes at least twice (e.g. at the beginning and end of each series). The beating frequency (129±4 beats/min, n = 188) was found to be stable over each series of experiments.

### Western blot analysis of prostanoid EP_1_ receptor expression

The membranes of the cultured rat cardiomyocytes were extracted with ice-cold lysis buffer (75 mM NaCl, 20 mM HEPES, 2.5 mM MgCl_2_, 0.1 mM EDTA, 0.1% Triton X-100, 10 µg/ml aprotinin, 20 mM glycerophosphate, 0.5 mM dithiothreitol, 0.1 mM sodium orthovanadate, 200 µg/ml phenylmethylsulphonyl fluoride and 1 mM sodium fluoride, pH 7.7). Samples were adjusted to the same protein concentration (determined using a BCA assay, Interchim, Montluçon, France). Aliquots of supernatant containing 20 µg of protein were boiled in sample loading buffer for 5 minutes and then loaded onto a 10% SDS-polyacrylamide gel and electrophoretically transferred to Immobilon-P membranes (Millipore, Saint Quentin en Yvelines, France). Non-specific binding sites on the membranes were blocked with 5% fat-free milk in 20 mM Tris-buffered saline (150 mM NaCl) plus 0.1% Tween-20 at pH 7.4 (TBST). The membranes were incubated in 5% fat-free milk in TBST containing a rabbit polyclonal anti-prostanoid EP_1_ receptor antibody (Cayman Chemicals, distributed by SPI-Bio, Massy, France) overnight at 4°C. The latter antibody had been raised against a synthetic peptide from the human prostanoid EP_1_ receptor (C-terminal amino acids 380–402; GLTPSAWEASSLRSSRHSGLSHF) conjugated to keyhole limpet haemocyanin. The antibody cross-reacts with rat prostanoid EP_1_ receptor (84% amino acid identity with its human counterpart) but does not bind to prostanoid EP_2_, EP_3_ or EP_4_ receptors. The membranes were then incubated with horseradish peroxidase-conjugated anti-rabbit IgG antibody in TBST for 1 h at room temperature. Lastly, immunoreactive bands were visualized with a chemiluminescence reagent (Amersham, France).

### Drugs

Prostanoids and synthetic analogues were all obtained from Cayman Chemicals (SPI-Bio): PGD_2_: [5Z,9α,11α,13E,15S]-9,15-dihydroxy-11-oxoprosta-5,13-dienoic acid; PGE_2_: methyl [5Z,9α,13E,15S]-11,15-dihydroxy-9-oxoprosta-5,13-dien-1-oic acid; PGF_2α_: [5Z,9α,11α,13E,15S]-9,11,15-trihydroxyprosta-5,13-dienoic acid; U-46619: 9,11-dideoxy-9α,11α-methanoepoxy prostaglandin F_2α_; 8-iso PGF_2α_: prosta-5,13-dien-1-oic acid, 9,11,15-trihydroxy-, (5Z,8β,9α,11α,13E,15S); BW245C: 4-imidazolidineheptanoic acid, 3-(3-cyclohexyl-3-hydroxypropyl)-2,5-dioxo-, (R*,S*); sulprostone: 5-heptenamide, 7-[3-hydroxy-2-(3-hydroxy-4-phenoxy-1-butenyl)-5-oxocyclopentyl]-N-(methylsulfonyl)-,[1R-1α(Z),2β(1E,3R*),3α]]-; misoprostol: 9-oxo-11α, 16-dihydroxy-16-methyl-prost-13E-en-1-oic acid, methyl ester; 11-deoxy PGE_1_: prost-13-en-1-oic acid, 15-hydroxy-9-oxo-, (13 E, 15 S); fluprostenol: 5-heptenoic acid, 7-[3,5-dihydroxy-2-(3-hydroxy-4-[3-(trifluoromethyl)phenoxyl]-1-butenyl]cyclopentyl]-, [1α(Z),2β(1E,3R*),3α,5α ]-; latanoprost: 9α, 11α, 15S-trihydroxy-17-phenyl-18,19,10-trinor-prost-5Z-en-1-oic acid; cloprostenol: 9α, 11α, 15R-trihydroxy-16-(3 chlorophenoxy)-17,18,19,20-tetranor-prost-5Z, 13E-dien-1-oic acid; butaprost: 9-oxo-11α,16R-dihydroxy-17-cyclobutyl-prost-13E-en-1-oic acid, methyl ester; iloprost: 5-((E)-[1S,5S,6R,7R]-7-hydroxy-6-[(E)-(3S,4RS)-3-hydroxy-4-methyl-1-octenyl]bicyclo[3.3.0]octan-3-ylidene)-pentanoic acid; beraprost: 2,3,3a,8b-tetrahydro-2-hydroxy-1-(3-hydroxy-4-methyl-1-octen-6-ynyl)-1H-cyclopenta[b]benzufuran-5-butanoic acid; SC-19220: 8-chloro-dibenz[b,f][1], [4]oxazepine-10(11H)-carboxy-(2-acetyl)hydrazide. PGD_2_, PGE_2_, PGF_2α_,8-isoPGF_2α_, BW245C, 11-deoxy PGE_1_, cloprostenol, beraprost and SC-19220 were supplied as crystalline powders, reconstituted with dimethyl sulfoxide (DMSO), stored at −20°C and further diluted in culture medium for use in experiments. U-46619, sulprostone, misoprostol, latanoprost, butaprost and iloprost were supplied as solutions in methylacetate. The methylacetate was evaporated and replaced by DMSO for storage at −20°C. Immediately prior to performance of the experiments, compound solutions in DMSO were diluted in culture medium. Fluprostenol was supplied as a solution in ethanol and then diluted in culture medium. The final concentrations of DMSO or ethanol never exceeded 0.2% and had no effect on the cardiomyocyte beating rates.

### Data processing and statistical analysis

All values correspond to the mean±the standard error of the mean (SEM). For each culture, differences in the beating rate were judged by comparing the mean frequency prior to addition of the compound and the peak frequency observed during the 21-minute period following addition of the compound (for the first method) or the 7-minute period following each concentration change (for the second method). Variations in the beating rate were evaluated in an analysis of variance (ANOVA) for repeated measures, followed by a Bonferroni-Dunn test. The threshold for statistically significance was set to p<0.05. Effects on the myocytes' beating rate were expressed as a percentage of the basal rate, “brady-arrhythmia” indicated a decrease of more than 50% and “tachy-arrhythmia” for an increase of more than 50% with chaotic, asynchronous, activity of the myocytes (as previously described by Li et al. [13].

The concentration-effect curves were analysed using non-linear regression implemented with GraphPad Prism software (version 5.01 from GraphPad Software Inc., San Diego, CA, USA). Sigmoidal curves were plotted to analyse the effects of agonists. The potency (pD_2_) of prostanoid receptor agonists was defined as the negative log_10_ of the agonist concentration achieving 50% of the maximal response (EC_50_) calculated by GraphPad Prism.

## Results

### Effects of prostanoid FP and IP receptor agonists

**Figure 1:**
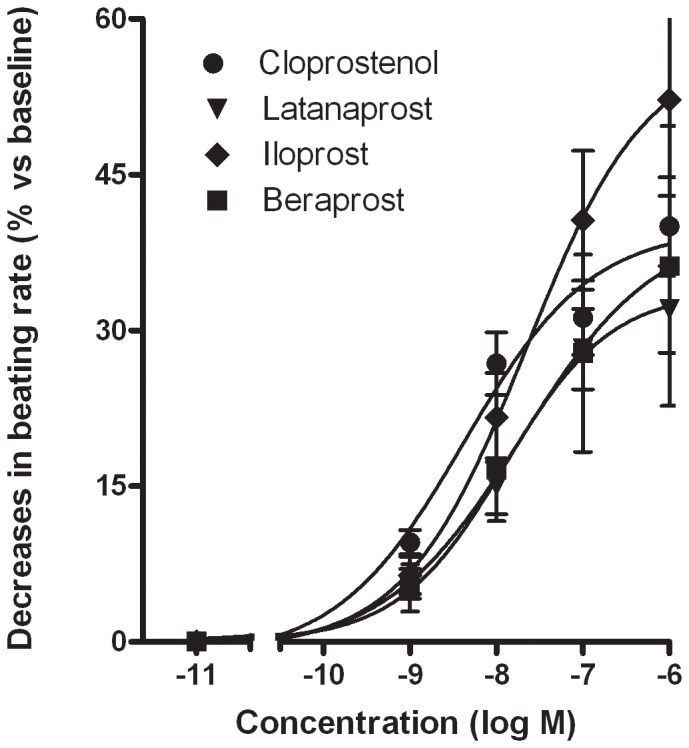
Effect of prostanoid IP and FP agonists on the cardiomyocyte beating rate. Cumulative concentration-response curves are shown for iloprost (♦, n = 5), beraprost (▪, n = 6), latanoprost (▾, n = 5) and cloprostenol (•, n = 5). Values are expressed as percent decrease (means±SEM from 5 or 6 experiments performed in duplicate).

At a concentration of 1 µM, PGF_2α_ and the prostanoid FP receptor agonists fluprostenol, latanoprost and cloprostenol produced a significant decrease in the myocytes' beating frequency (Table 1). Cumulative addition (1 nM to 1 µM) of cloprostenol or latanoprost induced a concentration-dependent decrease in the beating rate; the pD_2_ values were 8.4±0.3 (n = 5) and 7.9±0.3 (n = 5), respectively (Fig. 1). A cumulative, concentration-dependent experiment was not performed with the two weakest agonists (i.e. PGF_2α_ and fluprostenol).

**Table 1:** Effects of endogenous prostanoids and selective agonists on the beating rate of neonatal rat cardiomyocytes.

*Prostanoid agonists (1 µM)*	*Receptor selectivity ^a^*	*n*	*% change vs baseline*	*Brady- arrhythmia (n)*	*Tachy- arrhytmia (n)*
***FP agonists*** PGF_2α_					
	FP, EP_3_	7	−23±9 *	1	-
Fluprostenol	FP	7	−18±5 *	1	-
Latanoprost	FP	7	−34±5 *	2	-
Cloprostenol	FP	7	−41±9 *	2	-
***IP agonists***					
Iloprost	IP, EP_1_, EP_3_	7	−54±14 *	4	-
Beraprost	IP	7	−35±13 *	2	-
***DP agonists***					
PGD_2_	DP, FP, EP_3_	5	26±11 *	-	1
BW245 C	DP_1_	6	15±4 *	-	-
***TP agonist***					
U-46619	TP	5	25±15 *	-	2
***EP agonists***					
PGE_2_	EP, FP	12	3±5	-	-
Sulprostone	EP_1_>EP_3_	9	35±6 *	-	1
Butaprost	EP_2_	7	4±4	-	-
Misoprostol	EP_3_>EP_1,3,4_	6	8±5	-	-
11-deoxy-PGE_1_	EP_3_, EP_4_>EP_2_	5	7±2	-	-

* p<0.05; n is the number of independent preparations used; receptor selectivity as previously reported [4], [7], [8]. “bradyarrhythmia” indicated a decrease of more than 50%; “tachyarrhythmia” indicated an increase of more than 50% with chaotic, asynchronous, activity of the myocytes [13]; *n* is the number of preparations on which brady- or tachy-arrhythmia was observed.

At 1 µM, the prostanoid IP-receptor agonists iloprost and beraprost produced a marked reduction in the beating rate of the myocytes – indeed, in some experiments, the culture stopped beating and did not restart (Table 1). Prior to the beating arrest, the cardiomyocytes took on a stellate appearance and beat asynchronously. Cumulative addition (1 nM to 1 µM) of each of the compounds caused a concentration-dependent slowing of the rate; the pD_2_ values were 7.7±0.4 (n = 5) for iloprost and 7.7±0.7 (n = 6) for beraprost (Fig. 1).

### Effects of prostanoid DP and TP receptor agonists

At 1 µM, PGD_2_, BW245C (a specific prostanoid DP_1_ receptor agonist) and U-46619 (a stable TxA_2_ analogue selective for prostanoid TP receptor) produced significant increases in the myocytes' beating rate (Table 1). The isoprostane 8-iso-PGF_2α_ also acted as a specific prostanoid TP receptor agonist [15] at this concentration but was less potent than U-46619, since it increased the beating rate weakly and inconsistently (data not shown, n = 11). Tachyarrhythmia was observed with PGD_2_ and U-46619.

### Effects of prostanoid EP receptor agonists

At 1 µM, neither PGE_2_ (a non-selective agonist of the four prostanoid EP receptor subtypes), butaprost (a selective prostanoid EP_2_ receptor agonist), misoprostol (a prostanoid EP_2_ and EP_3_ receptor agonist) nor 11-deoxy-PGE_1_ (a prostanoid EP_2_, EP_3_, and EP_4_ receptor agonist) altered the spontaneous beating rate (Table 1). Since 11-deoxy-PGE_1_ is more selective for the three prostanoid EP receptor subtypes at lower concentrations [7], [8], we performed experiments (n = 4) at concentrations ranging from 1 nM to 1 µM. However, no significant effects were observed (data not shown).

**Figure 2:**
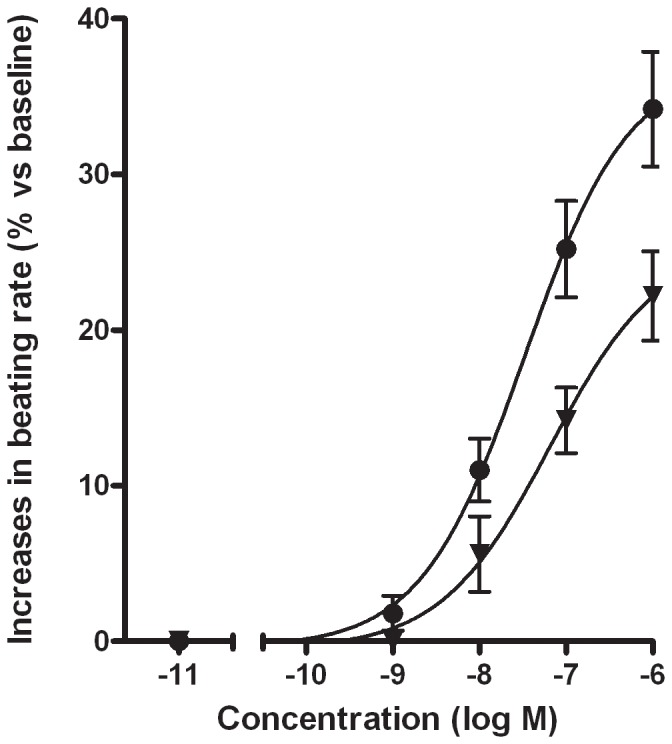
Effect of sulprostone on the cardiomyocyte beating rate. Cumulative concentration-response curves are shown for sulprostone in absence (•) or presence of 50 µM SC-19220 (▾), an antagonist at prostanoid EP1-receptor. Values are expressed as percent increase (means±SEM from 5 experiments performed in duplicate).

In contrast, the prostanoid EP_1_ and EP_3_ receptor agonist sulprostone (0.1–1 µM) rapidly (after 30 seconds) produced significant increases in the beating rate, with tachyarrhythmia occurring at 1 µM in 1 of the 9 experiments (Table 1). In three experiments, the cells were exposed to 5 µM of sulprostone without a further increase in the beating rate (data not shown). These effects were sustained throughout the experiments. Cumulative addition of sulprostone (1 nM to 1 µM) induced concentration-dependent increases in the beating rate of similar magnitude to those in the previous series, with a pD_2_ value of 7.4±0.5 nM (Fig. 2). Furthermore, pre-incubation of the cardiomyocytes with SC-19220 (50 µM) suppressed the sulprostone-induced (1 µM) increase in the beating rate by 34±8% (Fig. 2).

### Western blot analysis of prostanoid EP_1_ receptor expression

**Figure 3:**
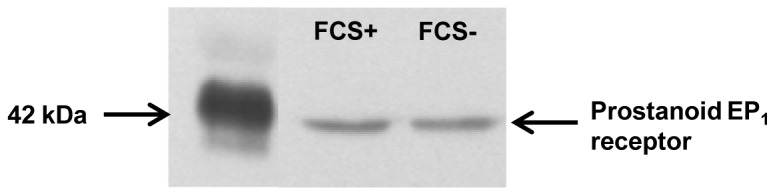
Western blot analysis of prostanoid EP_1_ receptor subtype in rat cardiomyocytes. Cells were cultured with 10% foetal calf serum (FCS+) for 3 days (as a control experiment) or then additionally incubated for 1 hour with 2% FCS and 1 µM indomethacin (FCS -), as performed in the functional experiments. The apparent mobility of the receptor was 42 kDa.

A Western blot analysis of prostanoid EP_1_ receptor expression at rat cardiomyocyte membranes (Fig. 3) clearly identified a 42-kDa band, which matched well with the predicted relative molecular mass of 43 kDa for the rat prostanoid EP_1_ receptor [7].

## Discussion

The main objective of the present study was to identify the prostanoid receptors involved in regulation of the spontaneous beating rate of neonatal rat cardiomyocytes. We chose to use mainly specific agonists for this purpose, given that (i) very few antagonists with a demonstrated specificity for rat prostanoid receptors are available and (ii) the pattern of responses with the various agonists was sufficient to provide evidence of the receptors' respective involvements.

### Effects of prostanoid FP and IP receptor agonists

A decrease in the beating rate was observed in presence of prostanoid FP and IP receptor agonists. Swiss 3T3 mouse fibroblast and A7r5 rat aorta smooth muscle cell lines [16], [17] are known to express only the prostanoid FP receptor; PGF_2α_, fluprostenol, cloprostenol and latanoprost are potent agonists in these cell lines, whereas iloprost (a prostanoid IP receptor agonist) is inactive. PGF_2α_ has been reported to increase the beating rate of rat cardiomyocytes and to produce tachyarrhythmias [13]. In addition to differences in the culture medium used to isolate and culture the cardiomyocytes (F-10 Nutrient Mixture (Ham) vs MEM), pre-treatment with indomethacin to avoid interference by the endogenous prostanoids may explain the contrasting effect observed with PGF_2α_ in our cardiomyocyte preparation. In line with our result with PGF_2α_, fluprostenol, cloprostenol and latanoprost induced a decrease in the neonatal rat cardiomyocytes' beating rate, with potencies similar to those reported in 3T3 and A7r5 cell lines. These data strongly suggest that prostanoid FP receptors negatively regulate the spontaneous beating rate of rat cardiomyocytes. This finding is in agreement with (i) the expression of prostanoid FP receptors in rat heart, (ii) the large quantity of prostanoid FP receptor mRNA in neonatal rat cardiomyocyte and (iii) the negative regulatory function attributed to this receptor [18]–[20]. PGI_2_ has previously been shown to induce a marked reduction in the contraction rate of rat cardiomyocytes at concentrations over 1 µM [13]. However, the compound is chemically unstable and also acts as an agonist of prostanoid TP and EP_3_ receptors [2]. It is therefore important to use selective, stable prostanoid IP receptor agonists to check that this type of receptor is indeed involved in negative regulation of the beating rate. Although, iloprost is more selective than PGI_2_ in most respects, the former compound exerts prostanoid EP_1_ and EP_3_ receptor agonist activity [2], [7], [8]. Beraprost is much more selective for prostanoid IP receptor as reported for cicaprost [8], [21]. Since iloprost and beraprost induced a marked reduction in the beating rate (with potencies similar to the potencies or binding affinities described in other preparations [8]), these results provide evidence of the involvement of prostanoid IP receptors. Prostanoid IP receptors have been detected by Western immunoblotting in isolated adult rat cardiomyocyte [22]. These results taken as a whole suggest that prostanoid IP and FP receptors negatively regulate the spontaneous beating rate in neonatal rat cardiomyocytes.

### Effects of prostanoid DP and TP receptor agonists

An increase in the beating rate was observed on addition of prostanoid DP and TP receptor agonists. PGD_2_ acts mainly through stimulation of prostanoid DP receptors. However, PGD_2_ binds to the cloned murine prostanoid FP receptor expressed in CHO cells [8] and also acts as a partial agonist in cell lines expressing either mouse or rat endogenous prostanoid FP receptors [16], [17]. Whatever the bindings of PGD_2_ on prostanoid FP receptors, the compound induced an increase in the cardiomyocyte beating rate. This finding suggests that prostanoid DP receptors positively regulate the spontaneous beating rate of rat cardiomyocytes. BW245C (a selective prostanoid DP_1_ receptor agonist that does not bind to the DP_2_ receptor and other prostanoid receptors [8], [16], [23]–[25]) also induced an increase in the beating rate; this observation provides further evidence that prostanoid DP_1_ receptors are involved in upregulation of the spontaneous beating rate in this cell type. Although the prostanoid DP_1_ and DP_2_ receptors are both expressed in rat heart [19], [23], prostanoid DP_2_ receptors do not appear to modulate the heart rate [26], [27]; this contrasts with our present finding for prostanoid DP_1_ receptors in rat cardiomyocytes and with the increase in the heart rate after administration to man of BW-245C [28], [29].

U-46619 is a highly potent, selective agonist of prostanoid TP receptors [2]. This finding suggests that functional TP receptors positively regulate the spontaneous beating rate in this preparation. The expression of prostanoid TP receptors in rat ventricular tissue has been confirmed [19]. An isomer of PGF_2α_ (8-iso PGF_2α_) has been shown to act on the TP receptor in various preparations but is much less potent than U-46619 [30], [31]; this may explain why the isomer of PGF_2α_ increased the cardiomyocyte beating rate to a less extent than U-46619 did.

### Effects of prostanoid EP receptor agonists

We applied PGE_2_ and various selective, synthetic prostanoid agonists in an attempt to identify the functional EP receptor subtypes in rat cardiomyocytes. PGE_2_ is a non-selective agonist of EP receptor subtypes but also has agonistic activity for FP receptors [8]. As previously described for experiments with the same preparation [13], PGE_2_ did not significantly alter the cardiomyocyte beating rate. However, the compound's lack of selectivity for prostanoid receptors may have masked positive or negative regulation of the beating rate in response to selective activation of EP receptor subtypes. At higher concentrations (2 to 10 µM), PGE_2_ reportedly produces tachyarrhythmia in neonatal rat cardiomyocytes [13]. Sulprostone is a potent, selective EP_1_ and EP_3_ receptor agonist but does not bind to other prostanoid EP receptors in the mouse and the rat [7], [8]. In our hands, the compound induced a marked increase in the beating rate. Butaprost is a selective prostanoid EP_2_ receptor agonist in both the rat and the mouse [7], [8]. Our finding that butaprost did not alter the spontaneous beating rate therefore rules out a regulating role for prostanoid EP_2_ receptors. Misoprostol is considered to be an agonist of prostanoid EP_2_ and EP_3_ receptors [2], [32], [33] but binds mainly to the EP_3_ receptor in the rat [7]. Misoprostol did not alter the beating rate of rat cardiomyocytes. Since the results with butaprost ruled out the involvement of prostanoid EP_2_ receptors, the data obtained with misoprostol further suggest that prostanoid EP_3_-receptors are also not involved in regulation of the beating rate of rat cardiomyocytes. 11-deoxy-PGE_1_ is an agonist of prostanoid EP_2_, EP_3_ and EP_4_ receptors [7], [8]. It has a 10- to 30-fold higher affinity for EP_3_ and EP_4_ receptors than for EP_2_ receptors [7], [8]. We found that 11-deoxy-PGE_1_ was devoid of any significant effect on the beating rate of rat cardiomyocytes, which again suggests that prostanoid EP_2_ and EP_3_ receptors (as well as EP_4_ receptors) are not involved in this process. The involvement of prostanoid EP_1_ receptors (relative to the other subtypes) was confirmed by the observed, inhibitory effect of SC-19220 (a specific antagonist on rat prostanoid EP_1_ receptors [6], [7]). We chose to use SC-19220 alone because other antagonists (such as AH6809, ONO-AE3-208 and ONO-8711) are non-specific EP antagonists in rat or only characterized in mouse preparations.

A previous study has reported that transcripts for all four prostanoid EP receptor subtypes were expressed by rat neonatal cardiomyocytes [34]. Three subtypes (EP_1_, EP_3_, EP_4_) have been detected by Western blotting in adult rat cardiomyocytes but the EP_1_ and EP_3_ receptors were the most abundant [22]. In the present study, the expression of prostanoid EP_1_ receptors on neonatal rat cardiomyocytes was confirmed at the protein level. These results provide persuasive evidence that rat heart predominantly expresses the prostanoid EP_1_ receptor subtype. Our results constitute the first demonstration that prostanoid EP_1_ receptors can positively regulate the beating rate of cardiomyocytes. In other respects, prostanoid EP_3_ and EP_4_ receptors are respectively involved in cardiomyocyte protection from oxidative stress [22], and PGE_2_-induced hypertrophic changes [34].

**Figure 4:**
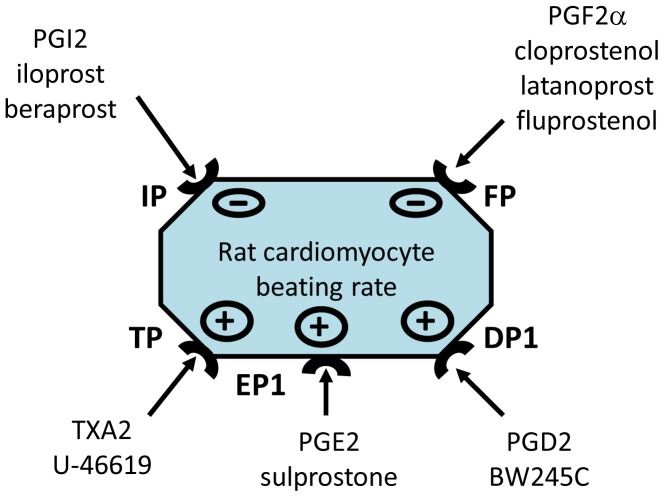
Modulation of the rat cardiomyocyte beating rate by prostanoid receptors. The spontaneous beating rate of neonatal rat cardiomyocytes is regulated negatively by prostanoid IP and FP receptors and positively by prostanoid TP, DP_1_ and EP_1_ receptors.

In conclusion, our results suggest that spontaneous beating rate of neonatal rat ventricular cardiomyocytes is negatively regulated by prostanoid IP and FP receptors and positively regulated by prostanoid TP, DP_1_ and EP_1_ receptors (Fig. 4). The synthesis of prostanoids in the heart is primarily determined by both the coronary vasculature (endothelium and vascular smooth muscle), and the cardiomyocytes [1], [9], [35]. It is difficult to estimate prostanoid concentrations in the myocardium from rates of release into the culture medium or coronary veins when variables such as prostanoid turnover are not known. In addition to PGE_2_ and PGF_2α_ (which are synthesized by the heart), PGI_2_ appears to be the predominant prostanoid released by rat ventricular cardiomyocytes, isolated rat or rabbit hearts or present in the blood of the human coronary sinus [1], [9], [36]. The direct effects of locally produced prostanoids on the beating rate of rat ventricular cardiomyocytes result from the balance between negative regulation by prostanoid IP and FP receptors and positive regulation by prostanoid TP, DP_1_ and EP_1_ receptors. The cell signalling mechanisms by which prostanoids might alter the electrical excitability of rat cardiomyocytes (e.g. the modification of ion channels) have, to the best of our knowledge, never been studied and thus require specific investigation. Since regulation of the beating rate also involves neurogenic, circulating, humoral factors or eicosanoids other than prostanoids, it is therefore even more complicated to explain or predict the *in vivo* outcome on the basis of *in vitro* data. However, cardiac synthesis of prostanoids is enhanced by the induction of hypoxia in isolated heart preparations or after *in situ* ischemia produced by coronary ligation [1]. Furthermore, prostanoid synthesis is known to be involved in the mechanism whereby ischemic preconditioning protects against reperfusion-induced tachyarrhythmia [11]. Cyclooxygenase (COX)-2 (the inducible form of COX) reportedly mediates the cardioprotective effects of the late phase of ischemic preconditioning by increasing the synthesis of prostanoids such as PGI_2_ and PGE_2_ [22], [37]. The cardioprotective effects of PGI_2_ have been ascribed to the opening of mitochondrial K(ATP) channels [22]. It is interesting to note that chronic treatment of normotensive and spontaneously hypertensive rats with iloprost reduced the incidence of reperfusion-induced arrhythmia [38]. In addition, COX-2-derived PGI_2_ has been found to mediate not only ischemic preconditioning but also isoflurane- and diazoxide-induced preconditioning *in vivo* [39]. On the whole, the cardiac prostanoids' effect is due (at least in part) to their regulatory actions on the cardiomyocyte beating rate.

## References

[1] Karmazyn M, Dhalla NS (1983) Physiological and pathophysiological aspects of cardiac prostaglandins. Can J Physiol Pharmacol 61: 1207–1225. 6362803 10.1139/y83-180

[2] Coleman RA, Smith WL, Narumiya S (1994) International Union of Pharmacology classification of prostanoid receptors: properties, distribution, and structure of the receptors and their subtypes. Pharmacol Rev 46: 205–229. 7938166

[3] Kennedy I, Coleman RA, Humphrey PP, Levy GP, Lumley P (1982) Studies on the characterisation of prostanoid receptors: a proposed classification. Prostaglandins 24: 667–689. 6131499 10.1016/0090-6980(82)90036-3

[4] Woodward DF, Jones RL, Narumiya S (2011) International Union of Basic and Clinical Pharmacology. LXXXIII: classification of prostanoid receptors, updating 15 years of progress. Pharmacol Rev 63: 471–538. 21752876 10.1124/pr.110.003517

[5] Abramovitz M, Adam M, Boie Y, Carriere M, Denis D, et al (2000) The utilization of recombinant prostanoid receptors to determine the affinities and selectivities of prostaglandins and related analogs. Biochim Biophys Acta 1483: 285–293. 10634944 10.1016/s1388-1981(99)00164-x

[6] Jones RL, Giembycz MA, Woodward DF (2009) Prostanoid receptor antagonists: development strategies and therapeutic applications. Br J Pharmacol 158: 104–145. 19624532 10.1111/j.1476-5381.2009.00317.x PMC2795261

[7] Boie Y, Stocco R, Sawyer N, Slipetz DM, Ungrin MD, et al (1997) Molecular cloning and characterization of the four rat prostaglandin E2 prostanoid receptor subtypes. Eur J Pharmacol 340: 227–241. 9537820 10.1016/s0014-2999(97)01383-6

[8] Kiriyama M, Ushikubi F, Kobayashi T, Hirata M, Sugimoto Y, et al (1997) Ligand binding specificities of the eight types and subtypes of the mouse prostanoid receptors expressed in Chinese hamster ovary cells. Br J Pharmacol 122: 217–224. 9313928 10.1038/sj.bjp.0701367 PMC1564924

[9] Oudot F, Grynberg A, Sergiel JP (1995) Eicosanoid synthesis in cardiomyocytes: influence of hypoxia, reoxygenation, and polyunsaturated fatty acids. Am J Physiol 268: H308–315. 7840277 10.1152/ajpheart.1995.268.1.H308

[10] Takayama K, Yuhki K, Ono K, Fujino T, Hara A, et al (2005) Thromboxane A2 and prostaglandin F2alpha mediate inflammatory tachycardia. Nat Med 11: 562–566. 15834430 10.1038/nm1231

[11] Arad M, Oxman T, Leor R, Rabinowitz B (1996) Prostaglandins and the antiarrhythmic effect of preconditioning in the isolated rat heart. Mol Cell Biochem 160–161: 249–255. 10.1007/BF00240056 8901480

[12] Birincioglu M, Olmez E, Aksoy T, Acet A (1997) The role of prostaglandin synthesis stimulation in the protective effect of captopril on ischaemia-reperfusion arrhythmias in rats in vivo. Pharmacol Res 36: 299–304. 9425619 10.1006/phrs.1997.0232

[13] Li Y, Kang JX, Leaf A (1997) Differential effects of various eicosanoids on the production or prevention of arrhythmias in cultured neonatal rat cardiac myocytes. Prostaglandins 54: 511–530. 9380795 10.1016/s0090-6980(97)00122-6

[14] Athias P, Frelin C, Groz B, Dumas JP, Klepping J, et al (1979) Myocardial electrophysiology: intracellular studies on heart cell cultures from newborn rats. Pathol Biol (Paris) 27: 13–19. 379749

[15] Kunapuli P, Lawson JA, Rokach JA, Meinkoth JL, FitzGerald GA (1998) Prostaglandin F2alpha (PGF2alpha) and the isoprostane, 8, 12-iso-isoprostane F2alpha-III, induce cardiomyocyte hypertrophy. Differential activation of downstream signaling pathways. J Biol Chem 273: 22442–22452. 9712868 10.1074/jbc.273.35.22442

[16] Griffin BW, Magnino PE, Pang IH, Sharif NA (1998) Pharmacological characterization of an FP prostaglandin receptor on rat vascular smooth muscle cells (A7r5) coupled to phosphoinositide turnover and intracellular calcium mobilization. J Pharmacol Exp Ther 286: 411–418. 9655886

[17] Griffin BW, Williams GW, Crider JY, Sharif NA (1997) FP prostaglandin receptors mediating inositol phosphates generation and calcium mobilization in Swiss 3T3 cells: a pharmacological study. J Pharmacol Exp Ther 281: 845–854. 9152393

[18] Adams JW, Migita DS, Yu MK, Young R, Hellickson MS, et al (1996) Prostaglandin F2 alpha stimulates hypertrophic growth of cultured neonatal rat ventricular myocytes. J Biol Chem 271: 1179–1186. 8557648 10.1074/jbc.271.2.1179

[19] Jovanovic N, Pavlovic M, Mircevski V, Du Q, Jovanovic A (2006) An unexpected negative inotropic effect of prostaglandin F2alpha in the rat heart. Prostaglandins Other Lipid Mediat 80: 110–119. 16846792 10.1016/j.prostaglandins.2006.05.014

[20] Ocklind A, Lake S, Krook K, Hallin I, Nister M, et al (1997) Localization of the prostaglandin F2 alpha receptor in rat tissues. Prostaglandins Leukot Essent Fatty Acids 57: 527–532. 9431817 10.1016/s0952-3278(97)90555-x

[21] Morrison K, Ernst R, Hess P, Studer R, Clozel M (2010) Selexipag: a selective prostacyclin receptor agonist that does not affect rat gastric function. J Pharmacol Exp Ther 335: 249–255. 20660124 10.1124/jpet.110.169748

[22] Shinmura K, Tamaki K, Sato T, Ishida H, Bolli R (2005) Prostacyclin attenuates oxidative damage of myocytes by opening mitochondrial ATP-sensitive K+ channels via the EP3 receptor. Am J Physiol Heart Circ Physiol 288: H2093–2101. 15604124 10.1152/ajpheart.01003.2004

[23] Shichijo M, Sugimoto H, Nagao K, Inbe H, Encinas JA, et al (2003) Chemoattractant receptor-homologous molecule expressed on Th2 cells activation in vivo increases blood leukocyte counts and its blockade abrogates 13,14-dihydro-15-keto-prostaglandin D2-induced eosinophilia in rats. J Pharmacol Exp Ther 307: 518–525. 12975488 10.1124/jpet.103.055442

[24] Walch L, Labat C, Gascard JP, de Montpreville V, Brink C, et al (1999) Prostanoid receptors involved in the relaxation of human pulmonary vessels. Br J Pharmacol 126: 859–866. 10193765 10.1038/sj.bjp.0702393 PMC1571232

[25] Woodward DF, Fairbairn CE, Krauss AH, Lawrence RA, Protzman CE (1995) Radioligand binding analysis of receptor subtypes in two FP receptor preparations that exhibit different functional rank orders of potency in response to prostaglandins. J Pharmacol Exp Ther 273: 285–287. 7714778

[26] Ishizuka T, Matsui T, Kurita A (2003) Ramatroban, a TP receptor antagonist, improves vascular responses to acetylcholine in hypercholesterolemic rabbits in vivo. Eur J Pharmacol 468: 27–35. 12729840 10.1016/s0014-2999(03)01626-1

[27] Johnston SL, Bardin PG, Harrison J, Ritter W, Joubert JR, et al (1992) The effects of an oral thromboxane TP receptor antagonist BAY u 3405, on prostaglandin D2- and histamine-induced bronchoconstriction in asthma, and relationship to plasma drug concentrations. Br J Clin Pharmacol 34: 402–408. 1467134 10.1111/j.1365-2125.1992.tb05649.x PMC1381468

[28] Al-Sinawi LA, Mekki QA, Hassan S, Hedges A, Burke C, et al (1985) Effect of a hydantoin prostaglandin analogue, BW245C, during oral dosing in man. Prostaglandins 29: 99–111. 3975430 10.1016/0090-6980(85)90155-8

[29] Orchard MA, Ritter JM, Shepherd GL, Lewis PJ (1983) Cardiovascular and platelet effects in man of BW 245C, a stable mimic of epoprostenol (PGI2). Br J Clin Pharmacol 15: 509–511. 6344895 10.1111/j.1365-2125.1983.tb02083.x PMC1427716

[30] Cracowski JL, Devillier P, Durand T, Stanke-Labesque F, Bessard G (2001) Vascular biology of the isoprostanes. J Vasc Res 38: 93–103. 11316945 10.1159/000051036

[31] Morrow JD, Hill KE, Burk RF, Nammour TM, Badr KF, et al (1990) A series of prostaglandin F2-like compounds are produced in vivo in humans by a non-cyclooxygenase, free radical-catalyzed mechanism. Proc Natl Acad Sci U S A 87: 9383–9387. 2123555 10.1073/pnas.87.23.9383 PMC55169

[32] Lydford SJ, McKechnie K (1994) Characterization of the prostaglandin E2 sensitive (EP)-receptor in the rat isolated trachea. Br J Pharmacol 112: 133–136. 8032634 10.1111/j.1476-5381.1994.tb13042.x PMC1910312

[33] Meja KK, Barnes PJ, Giembycz MA (1997) Characterization of the prostanoid receptor(s) on human blood monocytes at which prostaglandin E2 inhibits lipopolysaccharide-induced tumour necrosis factor-alpha generation. Br J Pharmacol 122: 149–157. 9298541 10.1038/sj.bjp.0701360 PMC1564914

[34] Miyatake S, Manabe-Kawaguchi H, Watanabe K, Hori S, Aikawa N, et al (2007) Prostaglandin E2 induces hypertrophic changes and suppresses alpha-skeletal actin gene expression in rat cardiomyocytes. J Cardiovasc Pharmacol 50: 548–554. 18030065 10.1097/FJC.0b013e318145ae2e

[35] Jenkins CM, Cedars A, Gross RW (2009) Eicosanoid signalling pathways in the heart. Cardiovasc Res 82: 240–249. 19074824 10.1093/cvr/cvn346 PMC2675928

[36] Mattera R, Stone GP, Bahhur N, Kuryshev YA (2001) Increased release of arachidonic acid and eicosanoids in iron-overloaded cardiomyocytes. Circulation 103: 2395–2401. 11352890 10.1161/01.cir.103.19.2395

[37] Bolli R, Shinmura K, Tang XL, Kodani E, Xuan YT, et al (2002) Discovery of a new function of cyclooxygenase (COX)-2: COX-2 is a cardioprotective protein that alleviates ischemia/reperfusion injury and mediates the late phase of preconditioning. Cardiovasc Res 55: 506–519. 12160947 10.1016/s0008-6363(02)00414-5 PMC3242376

[38] Tosaki A, Koltai M, Paubert-Braquet M (1990) Effect of iloprost on reperfusion-induced arrhythmias and myocardial ion shifts in isolated rat hearts. Eur J Pharmacol 191: 69–81. 1709406 10.1016/0014-2999(90)94097-h

[39] Alcindor D, Krolikowski JG, Pagel PS, Warltier DC, Kersten JR (2004) Cyclooxygenase-2 mediates ischemic, anesthetic, and pharmacologic preconditioning in vivo. Anesthesiology 100: 547–554. 15108967 10.1097/00000542-200403000-00013

